# Microbial Community Analysis in Sichuan South-road Dark Tea Piled Center at Pile-Fermentation Metaphase and Insight Into Organoleptic Quality Development Mediated by *Aspergillus niger* M10

**DOI:** 10.3389/fmicb.2022.930477

**Published:** 2022-06-27

**Authors:** Yao Zou, Ying Zhang, Yun Tian, Minqiang Liu, Yue Yuan, Yuqing Lai, Xuyi Liu, Wei Xu, Liqiang Tan, Qian Tang, Pinwu Li, Jingyi Xu

**Affiliations:** ^1^Department of Tea Science, College of Horticulture, Sichuan Agricultural University, Chengdu, China; ^2^Tea Refining and Innovation Key Laboratory of Sichuan Province, Chengdu, China; ^3^Tea Research Institute, Chongqing Academy of Agricultural Sciences, Chongqing, China

**Keywords:** microbial community, Sichuan South-road Dark Tea, organoleptic quality, *Aspergillus niger*, pile-fermentation

## Abstract

Microbes are critical in the Sichuan South-road Dark Tea (SSDT) organoleptic quality development during pile-fermentation. Piled tea center at fermenting metaphase is crucial for the conversion of its quality components. In this study, we investigated the microbial community of piled SSDT center below the stacked tea surface of 15 cm (SSDTB), 50 cm (SSDTX), and 85 cm (SSDTH) on the second turning time of pile-fermentation, respectively. Results showed that SSDTH and SSDTB had a higher similarity in the microbial community. *Pantoea* (36.8%), *Klebsiella* (67.7%), and *Aspergillus* (35.3%) were the most abundant in SSDTH, SSDTB, and SSDTX, respectively. We found 895 species were common among all samples, but 86, 293, and 36 species were unique to SSDTB, SSDTX, and SSDTH, respectively. *Aspergillus niger* showed high co-occurrence and was positively correlated with numerous microbes in SSDT samples, and *Aspergillus niger* M10 isolated from SSDTX was excellent at enhancing soluble sugar (SS), amino acids (AAs), theaflavin (TF), and thearubigins (TR) contents, while decreasing catechin (Cat), tea polyphenols (TPs)/AA, Caf/SS, Cat/SS, TPs/SS, and (TPs + Caf)/SS levels in AM10 post-fermentation, as compared with the control. Moreover, it also produced a noticeable difference in the CIELab parameters in dried, liquor, and infused tea colors between AM10 and control during fermentation. When it was further inoculated on differential mediums, we detected glycoside hydrolases, namely, β-glucosidase, mannosidase, pectinase, cellulase, amylase, and α-galactosidase being secreted by *Aspergillus niger* M10. Taken together, SSDXT presented a more unique microbial community. *Aspergillus niger* M10 probably improved the sweet and mellow taste, and the yellow brightness and red color of SSDT during fermentation. It also provided new insights into the microbial profile and organoleptic quality development mechanism of SSDT during pile-fermentation.

## Introduction

Sichuan South-road Dark Tea (SSDT), the staple product of Sichuan Dark Tea, only produced in the Ya’an district, is a popular beverage among the Tibetan communities of southwest China (Supplementary Figure 1). SSDT is usually pressed into bricks, and it has a brownish red to blackish brown appearance, bright yellowish red liquor color, mellow and sweet taste, and a unique pure aroma. For a Tibetan, it is not only a life necessity but also a folk medicine. While for the residents of inland China, it is a well-known health drink with hypolipidemic activities (Zou et al., 2020).

Pile-fermentation is an essential procedure for accelerating the transformation of chemical components which are responsible for the unique flavor of dark tea (Zou, 2014). During the transformation process, microorganisms play an important role *via* microbial metabolism, natural oxidation, and extracellular enzyme activities (Zhang et al., 2016). To find out the specifics of microbial participation in the dark tea quality development, Li et al. (2018) explored the microbial community of Pu-erh tea during their natural solid-state fermentation and found that *Aspergillus* was the main flavor-producing microorganism during early fermentation, while *Bacillus*, *Rasamsonia*, *Lichtheimia*, and *Debaryomyces* were producing flavors during late fermentation. Besides, Li et al. (2020) also discovered that *Aspergillus*, *Candida*, *Debaryomyces*, *Penicillium*, unclassified_k_Fungi, and unclassified_o_Saccharomycetales participated in the volatiles metabolism of Fuzhuan tea during its processing. Researchers discovered that the microbial community presented during pile-fermentation was responsible for the dark tea organoleptic quality development (Zhu et al., 2020; Lin et al., 2021), but it was easily affected by the temperature, humidity, and natural inoculum sources of the environment. Thus, Chinese dark tea produced in different areas exhibited its own variations in the microbial community, taste-active components, and organoleptic quality (Zhu et al., 2020). Compared with the reports about microbes and the quality of Pu’erh, Fuzhuan, and Liubao tea, there has been little research on SSDT. To date, the relationship between microbial community and organoleptic quality of SSDT remains poorly understood. Turning is a necessary process of pile-fermentation, which ensures tea quality uniformity, and is considered the criteria for evaluating the piling period. Usually, the pile-fermentation period and piled tea location will influence the microbial community composition. Yan et al. (2021a,b) verified that the microbial community of the Sichuan Dark Tea varied with the piling stage, as *Aspergillus*, *Enterobacteriaceae*, *Pseudomonas*, and *Bacillus* were each abundant in the different piling periods. Besides that, Xiong (2017) found that the similarity of the microbial community in piled SSDT center between the second turning time and the other periods did not exceed 12%. Coincidentally, Zou (2014) detected the significant changes in tea taste-active components at piled SSDT center during this stage, and after this time, SSDT’s quality transformation has almost completed (Qi, 2011; Zou, 2014; Xiong, 2017). Hence, the second turning time, generally taken as the metaphase of SSDT piled-fermentation, is crucial for SSDT quality formation. The unique microbial community and specific functional microbes presented in piled SSDT center at this time may play an important role in accelerating quality-active components conversion.

Thus, in this work, we not only analyzed the microbial community in different locations of the piled SSDT center during the second turning period of pile-fermentation but also investigated how the core microbe isolated from piled SSDT center affects the taste and color quality of SSDT. This comprehensive study may help decode the organoleptic quality development mechanism of SSDT during pile-fermentation.

## Materials and Methods

### Samples Preparation, Shotgun Metagenomic Sequencing, and Data Analysis

Sichuan South-road Dark Tea (SSDT) undergoing pile-fermentation, was collected from Helong Tea Co. Ltd. (Ya’an, China) in 2018. The samples were separately collected from piled tea center line 15 cm (SSDTB), 50 cm (SSDTX), and 85 cm (SSDTH) below the tea pile surface on the second turning time (Supplementary Figure 2A), after which they were immediately transported to the laboratory on dry ice and finally stored at −80°C for further microbial shotgun metagenomic analysis.

The microorganisms in the SSDT samples were collected by differential centrifugation, and their genomic DNA was extracted using the DNeasy^©^ PowerSoil^©^ Kit (QIAGEN, Germany) as per the manufacturer’s instructions. The metagenomic DNA libraries were constructed using the NEBNext^®^ Ultra™ DNA Library Prep Kit for Illumina (New England Biolabs, Ipswich, MA, United States). After evaluating the libraries’ quality, sequencing was performed using Illumina MiSeq according to the manufacturer’s protocol at the Basebio Company (Chengdu, China). After splicing, filtering, and quality cutting, the obtained reads were subjected to classification analysis. Sequences with the same taxonomic annotation were classified into an Operational Taxonomic Unit (OTU), and a taxonomic analysis of the OTUs was conducted using the RDP classifier Bayesian algorithm at a 97% identity threshold. The community composition and scientific classification of each sample were established at the kingdom, phylum, class, order, family, genus, and species levels (Chen et al., 2016). The α-diversity of each sample was calculated by using the Chaol and Shannon indices to evaluate the abundance and diversity of the microbial communities in the different SSDT samples (Rogers et al., 2016). R tool was used to conduct clustering analysis, draw heatmaps, establish a community structure histogram, and construct the Venn diagram displaying the common and unique species in each sample (Fouts et al., 2012). Python software was used to carry out the *t*-test for pairwise comparisons between samples based on microbial relative abundance.

### Effect of Core Microbe on Sichuan South-road Dark Tea (SSDT) Organoleptic Quality Development

#### Inoculation and Fermentation

*Aspergillus niger* M10 (NCBI ID: KX507070; Supplementary Figure 2B) isolated from SSDTX by us was cultured on the potato dextrose agar medium at 28°C for 5 days for spore germination. The spore suspension was obtained by washing the agar surface with 0.9% of sodium chloride solution, and then adjusted using sterile distilled water, to a final concentration of 10^5^ cfu/ml.

Maozhuang tea (raw material of SSDT) liquor for submerged fermentation was prepared according to Zou’s method (2014), after being autoclaved at 121°C for 20 min. A part of this was inoculated with the spore suspension (2%, v/v) as M10 treatment, while the others were inoculated with the sterile distilled water as the control. All the treatments were fermented at 60°C for 20 days, with the shaker speed at 120 rpm.

Regarding solid-state fermentation, before inoculating, Maozhuang teas’ water content was adjusted to 25%, then they were put into the triangular flask with air-vent capping (50 g per flask), and finally sterilized. All the sterilized samples were divided into two groups: one group (AM10) was inoculated with the spore suspension (5%, v/m), while the other group used equivalent sterile distilled water as the control. After mixing evenly, all the samples were fermented for 20 days at 60°C in the constant temperature and humidity incubator (GZ-120-HSH, Guangzhi, China).

The experiments were performed in triplicates, and during fermentation, sampling was conducted every two days for chemical analysis or CIELab parameters detection.

#### Chemical Analysis and CIELab Parameters Detection

The water extract content (AE) of the sample was determined according to the China National Standard GB/T8305-2013. The contents of tea polyphenols (TPs), amino acids (AAs), caffeine (Caf), and catechin (Cat) were measured according to GB/T8313-2008, GB/T8314-2013, GB/T8312-2013, and GB/T8313-2008, respectively. Soluble sugar (SS) and chlorophyll (Chl) were separately quantified using anthrone-sulfuric acid colorimetry and ether colorimetry (Zou, 2014), while theaflavin (TF), thearubigins (TR), and theabrownin (TB) were measured using spectrophotometric methods (Huang, 1997). Tea pigment (Tpi) = TF + TB + TR. Furthermore, CIELab color parameters of dried, liquor, and infused teas, and the derivative parameters of which were determined as described by Zou et al. (2020).

#### Determination of Glycoside Hydrolase Secreted by *Aspergillus niger* M10

Differential media was used to screen glycoside hydrolase secreted by *Aspergillus niger* M10, of which cellulase, pectinase, β-glucosidase, mannosidase, amylase, and α-galactosidase were identified according to the method of Hu (2020), Cheng et al. (2020); Hu et al. (2011), Tian et al. (2020); He et al. (2019), and Zhang (2018), respectively. The hydrolytic ring diameter was quantified as per Liu (2015).

### Statistical Analysis

Principal component analysis (PCA) and cluster analysis of chemical components and color parameters were carried out using Origin 2021 (Origin Lab Corporation, MA, United States).

## Results

### Microbial Community Profile in Piled Sichuan South-road Dark Tea (SSDT) Center at Pile-Fermentation Metaphase

**FIGURE 1:**
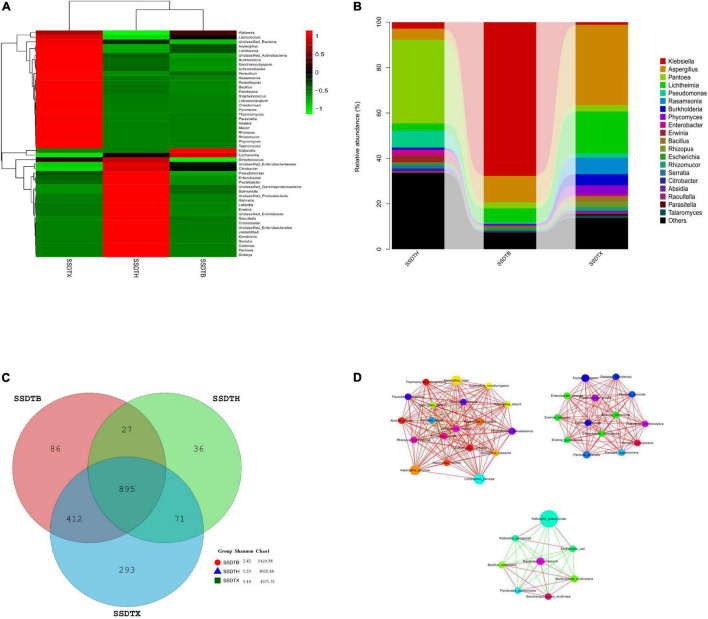
Microbial profile of piled Sichuan South-road Dark Tea (SSDT) center at pile-fermentation metaphase. **(A)** Cluster analysis heatmap based on the abundance of the top 50 genera; abundance changes of different genera in each sample are displayed by color gradient of the color block, and the scale bar shows the variation range of the normalized abundance of the genera. **(B)** Histogram of the relative abundance of the top 20 genera; various colors represent different genera, and the relative abundance of each genus is represented by the length of the column. **(C)** Venn diagram; various colors represent the different samples, and numbers represent the detected species in the SSDT samples. **(D)** Correlation network of the top 50 dominant species; colorful nodes represent the different species, node size is proportional to their abundance, while the red solid line and the green solid line indicate the positive association and negative association between species, respectively.

We conducted clustering analysis and generated the heatmap to exhibit the similarity in microbial community composition between different samples (Figure 1A), and also presented the 20 most frequent populations in different samples (Figure 1B), it showed that SSDTH and SSDTB had a higher similarity in the microbial community, and *Pantoea* (36.8%) was the most abundant in SSDTH, and followed by the unclassified *Enterobacterales* (14%). Whereas, in SSDTB and SSDTX, the abundance of *Pantoea* decreased to 2.7 and 2.9%, respectively. *Klebsiella*, with a higher abundance of 67.7%, was discovered in SSDTB, while the abundance of *Aspergillus* was also up to 11.6%. Nevertheless, the abundance of *Klebsiella* declined to 1.1% in SSDTX, whereas that of *Aspergillus* increased to 35.3%. Undoubtedly, *Aspergillus* was the most dominant genus in SSDTX, followed by *Lichtheimia* with a relatively high abundance of 18.5%. Besides, we found 895 species were common among all the samples, but 86, 293, and 36 species were unique to SSDTB, SSDTX, and SSDTH, respectively. Moreover, SSDTB and SSDTX shared 1,307 species, SSDTX and SSDTH shared 966 species, while SSDTH and SSDTB shared 922 species. Furthermore, Chao1 of SSDTH, SSDTB, and SSDTX were 1,029.48, 1,420.58, and 1,671.31, respectively, while their Shannon were 5.23, 2.42, and 5.14, respectively (Figure 1C). Obviously, SSDTX had the highest species richness and relatively higher diversity, while SSDTB and SSDTH exhibited the lowest species diversity and richness, respectively. In addition, SSDTB-SSDTH showed a significant difference in microbial abundance in 17 phyla, 422 genera, and 1,145 species. SSDTB-SSDTX also showed it in 32 phyla, 665 genera, and 1,573 species, while SSDTH-SSDTX exhibited it in 27 phyla, 647 genera, and 1,571 species. Moreover, we calculated the Spearman correlation coefficient between each of the top 50 dominant species in terms of abundance, with the Mothur software, and found that the correlation network between the dominant species was selected by the criteria of | rho| > 0.8 and *p* < 0.01. The result was visually depicted using the Cytoscape software^1^. *Aspergillus* was associated with plenty of microbes, of which *Aspergillus niger* showed high co-occurrence and was positively correlated with numerous microbes in the SSDT samples (Figure 1D), indicating that *Aspergillus niger* may have positively cooperated with other strains and also played an important role in the conversion of SSDT quality components during pile-fermentation.

### Effect of *Aspergillus niger* M10 on Sichuan South-road Dark Tea (SSDT) Taste-Active Components During Fermentation

**FIGURE 2:**
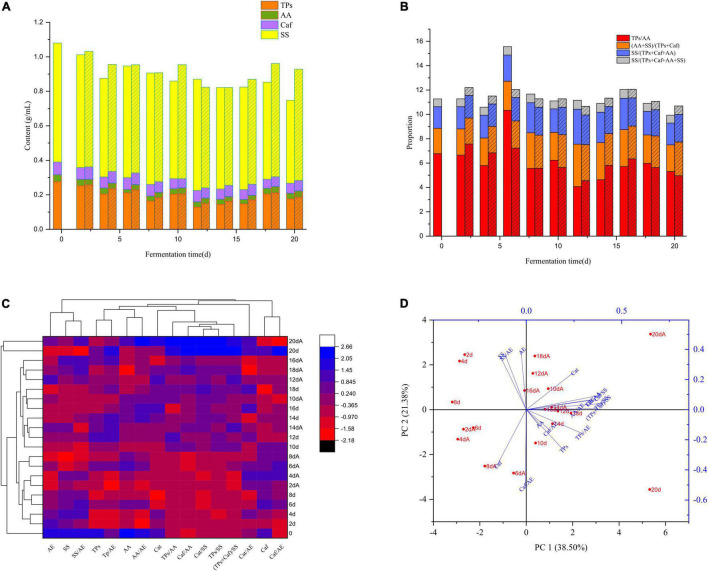
Effect of *Aspergillus niger* M10 on the SSDT taste quality during fermentation. **(A)** Changes of primary taste-active components during submerged fermentation; column represents control and column filled with diagonal reflects M10. **(B)** Changes of the ratios of primary taste-active components during submerged fermentation; column reflects control and column filled with diagonal reflects M10. **(C)** Changes of sample taste-active components during solid-state fermentation; each row and column in the heatmap represents the samples at different fermentation times and a taste-active component, respectively; the red and blue colors denote their levels. The fermentation time marked with A represents AM10, while the unmarked represents the control. **(D)** PCA of samples undergoing solid-state fermentation based on the taste-active components.

We isolated *Aspergillus niger* M10 from SSDTX, and undertook both submerged and solid-state fermentation to investigate its effect on SSDT taste-active components transformation. It was found that the contents of the primary taste-active ingredients and their proportions varied drastically post submerged fermentation. The levels of TPs, AA, Caf, SS, and TPs/AA in the control at 20 d decreased by 36.27, 18.66, 20.36, 30.34, and 21.64%, respectively, as compared with tea liquor at 0 d, while those in the M10 reduced by 32.82, 8.28, 17.12, 6.58, and 26.75%, respectively (Figures 2A,B). Moreover, in stark contrast to 0 d liquor, the levels of (AA + SS)/(TPs + Caf), SS/(TPs + Caf + AA), and SS/(TPs + Caf + SS + AA) in the control and M10 samples at 20 d increased by 26.32 and 32.37%, 26.49 and 28.48%, and 7.5 and 8.71%, respectively (Figure 2B). Notably, after fermentation (20 d), *Aspergillus niger* M10 enhanced the levels of SS, (AA + SS)/(TPs + Caf), and SS/(TPs + Caf + AA) in M10 by 34.11, 26.32, and 26.49%, respectively, with slightly increased TPs, AA, and Caf contents as compared with the control (Figures 2A,B). TPs and Caf are the main indicators of astringency and bitterness attributes, while AA and SS indicate the umami and sweetness attributes of tea. The above results suggested that *Aspergillus niger* M10 could directly or indirectly alter the ratio of umami and sweet components’ levels to the bitterness ingredients’ levels, while simultaneously alleviating the decline of taste-active components’ levels induced by fermentation, thereby resulting in improved thickness and sweetness attributes of SSDT liquor.

Besides, we generated the heatmap and PCA plot diagram based on the taste-active components detected in solid-state fermentation (Figures 2C,D). It clearly showed that levels of taste-active components of different samples fluctuated with the progress in fermentation. We clustered all the samples into 3 categories at a Euclidean distance of 7.67, of which samples of 20 d and 20 dA (A represented the sample in AM10) were clustered into one category, the sample of 0 d was a single category, while the remaining samples were attributed to one category (Figure 2C). This implied that after fermentation, the tea taste-active components’ levels noticeably differed from that of Maozhuang tea. Further analysis indicated that although samples of 20 d and 20 dA were clustered into one category, they were distant from each other in Figure 2D. The sample of 20 d was mainly affected by the TPs and TPs/AA levels, while the 20 dA sample was mainly influenced by the TPs/SS, (TPs + Caf)/SS, and Caf/SS levels, respectively (Figure 2D). Besides, the levels of AA and SS in the 20 dA sample were enhanced by 32.83 and 98.43%, respectively, whereas the Cat, TPs/AA, Caf/SS, Cat/SS, TPs/SS, and (TPs + Caf)/SS levels, respectively, declined by 28.84, 31.52, 57.36, 64.47, 54.9, and 55.49%, as compared with that of the 20 d sample (Figure 2C). We demonstrated that the taste-active components representing the bitterness and astringent attributes of SSDT fermented by *Aspergillus niger* M10 diminished, but the ones reflecting sweetness were reinforced, significantly with the proportion of TPs or Cat level to SS level prominently decreasing. Therefore, this shows that *Aspergillus niger* M10 has an excellent ability to reduce the bitterness and increase the sweetness attributes of SSDT taste during pile-fermentation.

### Changes in Sichuan South-road Dark Tea (SSDT) Color Components Mediated by *Aspergillus niger* M10 During Solid-State Fermentation

**FIGURE 3:**
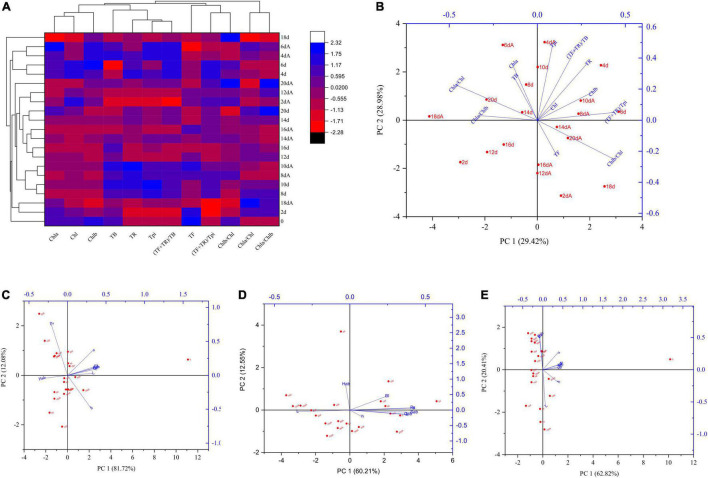
Influence of *Aspergillus niger* M10 on SSDT color quality during fermentation. **(A)** Changes of sample color components during solid-state fermentation; each row and column in the heatmap represents samples at different fermentation times and a color component, respectively; the fermentation time marked with A represents AM10, while those unmarked represent the control. **(B)** PCA of samples undergoing solid-state fermentation based on color components. **(C)** PCA of dried tea CIELab parameters of different samples. **(D)** PCA of liquor tea CIELab parameters of different samples. **(E)** PCA of infused tea CIELab parameters of different samples.

Solid-state fermentation mediated by *Aspergillus niger* M10 had a greater effect on SSDT color components. In terms of primary pigments, whose levels in different samples fluctuated during fermentation, the Euclidean distance of 20 d and 20 dA samples was close, but the TF and TR contents of the 20 dA sample increased by 62.26 and 11.71%, respectively, as compared with the 20 d sample (Figure 3A). Noticeably, the 20 d sample was primarily affected by Chla/Chl, Chla, and TB levels, while the 20 dA sample was mainly affected by the Chlb/Chl and TF levels (Figure 3B). Usually, TB, TF, and TR contribute to the reddish-brown color, yellow brightness, and the red tea infusion (Wan, 2020; An et al., 2021). The above results suggested that *Aspergillus niger* M10 probably could improve the yellow brightness and red degree of SSDT color. Simultaneously, *Aspergillus niger* M10 also significantly affected the color parameters of dried tea, tea liquor, and infused tea during fermentation. With regard to dried tea, the 20 dA sample was mainly affected by Ps level and had the lowest PC1 score but the highest PC2 score, while the 20 d sample obtained 0 level of PC1 score and a relatively higher PC2 score, and was mainly influenced by Cab and BI levels (Figure 3C). With respect to tea liquor color, the 20 dA sample had a relatively high PC1 score but a low PC2 score, which was mainly affected by Sab, Cab, and b levels, whereas the sample of 20 d obtained a slightly lower PC1 and PC2 score, as it was primarily influenced by the L level (Figure 3D). In addition, in regard to infused tea, the 20 dA sample showed a relatively higher PC1 score, but a lower PC2 score than that of the 20 d sample, since it was mainly affected by the L and h levels (Figure 3E).

### Glycoside Hydrolases Secreted by *Aspergillus niger* M10

**FIGURE 4:**
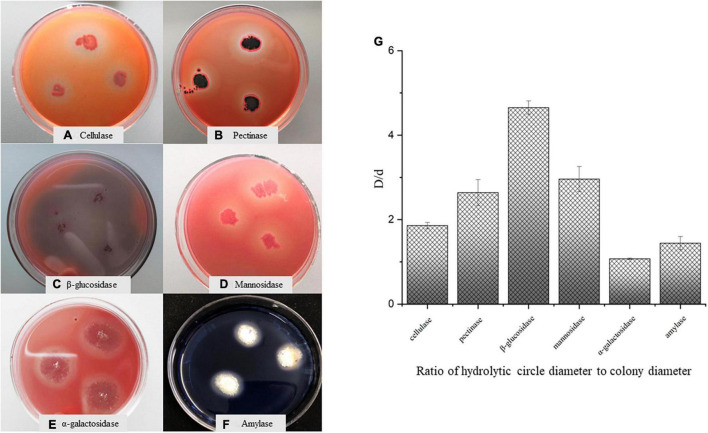
Analysis of glycoside hydrolase secreted by *Aspergillus niger* M10. **(A)** Cellulase, **(B)** pectinase, **(C)** β-glucosidase, **(D)** mannosidase, **(E)** α-galactosidase, **(F)** amylase, and **(G)** ratio of hydrolytic diameter to colony diameter.

The raw materials of SSDT are very rough and old, indicating a usually high cellulose level but being low in SS level. Whereas after fermentation, *Aspergillus niger* M10 evidently promoted SS level in the tea sample as compared with the control, suggesting that this strain may secrete some glycoside hydrolases to accelerate the macromolecular carbohydrate degradation. Thus, we used the differential medium to detect the glycoside hydrolases secreted by *Aspergillus niger* M10. Consequently, we found that *Aspergillus niger* M10 produced hydrolysis circles on the differential medium and could secrete cellulase, pectinase, β-glucosidase, mannosidase, α-galactosidase, and amylase (Figures 4A–F). The diameters of the hydrolytic circles of various glycoside hydrolases were in the following order: β-glucosidase>mannosidase>pectinase>cellulase>amylase> α-galactosidase (Figure 4G).

## Discussion

Usually, the different locations of piled SSDT during pile-fermentation encountered different sets of temperature and humidity, which affected the growth and reproduction of microbes, and finally produced the differences of microbial community composition in SSDTB, SSDTX, and SSDTH, respectively. The distinct wet climate in the Ya’an district and its resultant characteristic microflora in the air and fresh tea leaves probably led to the apparent differences in microbial community composition between SSDT and the other dark teas (Lin et al., 2021). The poor individual distribution uniformity of microbes in SSDTB may result from *Klebsiella* thriving in the high-humidity and optimum temperature environment. Although certain *Klebsiella* species are pathogenic, their abundance could be easily mitigated by high temperature, and consequently, we detected a noticeable decline of *Klebsiella* in SSDTX (high-temperature location). However, we discovered an overwhelming dominance of *Aspergillus* in SSDTX, parts of which may be thermophilic microorganisms (Copetti et al., 2011). Previous research suggested that thermophilic fungi probably could sporulate abundantly and also maintain the long-term viability of their spores in high temperatures, thus allowing them to multiply in harsh environments (Mao et al., 2017).

**FIGURE 5:**
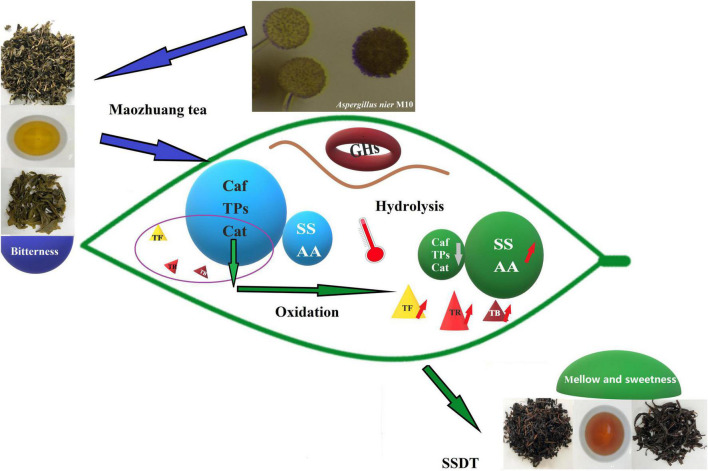
A schematic diagram summarizing the specifics of *Aspergillus nier* M10 mediating SSDT taste and color quality. Changes in the main indicators of taste and color are listed. Orange arrows represent increase and gray arrows represent decrease.

*Aspergillus* was the predominant genus in various dark teas and was involved in their component’s conversion process (Li et al., 2018, 2020; Lin et al., 2021). Among them, *Aspergillus niger* was speculated to be vital in FBT and Pu’erh tea quality development (Zhang et al., 2016; Rui et al., 2019). In this study, the distinct decrease in levels of typical taste-active ingredients of tea samples post-fermentation as compared to the raw materials was consistent with that in other dark teas (Zhao et al., 2015; Mao et al., 2017; Cheng et al., 2020; Feng et al., 2020; Cheng et al., 2021), and such degradation probably resulted from a synergistic effect of damp heat and microbial activity. Microbial fermentation usually produces a battery of extracellular enzymes catalyzing a wide variety of reactions (Zhu et al., 2020). This is evident from the distinct decline of Cat and the sharp increase of SS and AA in AM10 as compared with the control post-fermentation, possibly due to hydrolysis, oxidation, and polymerization mediated by *Aspergillus niger* M10 *via* its extracellular polyphenol oxidase, catalase, tannase, and other glycoside hydrolases (Zhao et al., 2015; Zhu et al., 2016; Hu et al., 2021). Particularly, the significant increase of SS as compared with control, implied that *Aspergillus niger* M10 possibly is crucial in the degradation of complex carbohydrates in tea. We detected several glycoside hydrolases secreted by *Aspergillus niger* M10 in this study, which may actively degrade the cellulose mass resulting from old and rough raw materials of SSDT. Previous studies based on the association analysis of metabonomics and high-throughput sequencing speculated that *Aspergillus* produced abundant hydrolytic enzymes to degrade cellulose, pectin, and protein substances in the cell wall of tea leaves to form SS, AAs, soluble pectin, and other compounds, thereby making a greater contribution to the mellow and sweet taste of Pu’erh tea (Wang et al., 2011; Li et al., 2018). While in this study, we isolated *Aspergillus niger* M10 from SSDTX, and directly verified that solid-state fermentation could reduce the main taste-active ingredients’ levels and make the liquor taste mellow, whereas *Aspergillus niger* M10 aggravated the reduction of the typical bitterness component (Cat), but dramatically increased the sweetness component (SS), which altered the ratio of bitterness components’ levels to the sweet and umami components’ levels, resulting in a more pure and sweet taste. Beyond that, we detected a significant increase of both TF and TR as compared with the control, in AM10 samples post-fermentation, indicating that *Aspergillus niger* M10 probably mediates the brightness and yellowish-red color of SSDT liquor by affecting the conversion of TF and TR (Figure 5). In contrast to Pu’erh tea, TB is the pigment behind the infusion color (Cheng et al., 2021), while TF and TR apparently are more important to the SSDT liquor color. Furthermore, *Aspergillus niger* M10 was isolated from a high-temperature location of piled SSDT center and was inoculated on Maozhuang tea and conducted solid-state fermentation at high temperature, indicating that it might be a thermotolerant strain. Moreover, in this work, we expect to ascertain the effect of specific functional microbe on SSDT quality to facilitate the application of which in SSDT production, and we will continue to explore the effects of all major microorganisms on the sensory quality of SSDT utilizing metabolomics analysis in our subsequent work.

## Conclusion

In this work, we systematically revealed the microbial profile of different locations of piled SSDT center during the crucial pile-fermentation stage and verified the influence of specific functional microbe isolated from piled SSDT center on the organoleptic quality formation of SSDT. We found that SSDTH and SSDTB had a higher similarity in the microbial community, but SSDTX exhibited the maximum unique species and with *Aspergillus* (35.3%) as the most abundant genus. *Aspergillus niger* M10 isolated from SSDTX mediated the SSDT taste and color quality *via* altering the bitterness and sweetness active components and their ratio, combined with the yellow brightness, red color, and CIElab parameters during pile-fermentation. These results suggest that the piled SSDT center is an important resource of functional microbes and needs to be further researched, besides that, the results also pave the way for the further applications of functional microbes in SSDT production.

## Data Availability Statement

The datasets presented in this study can be found in online repositories. The names of the repository/repositories and accession number(s) can be found below: https://www.ncbi.nlm.nih.gov/, PRJNA828626.

## Author Contributions

YZo, WX, and QT designed the experiments. YZh, YY, ML, YT, XL, and YL performed the experiments. YZo, LT, JX, and PL analyzed the data. YZ and YZ wrote the manuscript. All authors contributed to the article and approved the submitted version.

## Conflict of Interest

The authors declare that the research was conducted in the absence of any commercial or financial relationships that could be construed as a potential conflict of interest.

## Publisher’s Note

All claims expressed in this article are solely those of the authors and do not necessarily represent those of their affiliated organizations, or those of the publisher, the editors and the reviewers. Any product that may be evaluated in this article, or claim that may be made by its manufacturer, is not guaranteed or endorsed by the publisher.

## Footnotes

1
http://www.cytoscape.org

## Supplementary Material

Supplementary Figure 1 Sichuan South-road Dark Tea.

Supplementary Figure 2 Sampling schematic diagram and *Aspergillus niger* M10 strain isolated from SSDTX. **(A)** Sampling point. **(B)** Morphological characteristics of *Aspergillus niger* M10.
