# Supplementary figures and images for: Microbial Community Analysis in Sichuan South-road Dark Tea Piled Center at Pile-Fermentation Metaphase and Insight Into Organoleptic Quality Development Mediated by Aspergillus niger M10
Source: Front Microbiol. 2022 Jun 27;13:930477. doi: 10.3389/fmicb.2022.930477 (PMC9272892; doi:10.3389/fmicb.2022.930477)

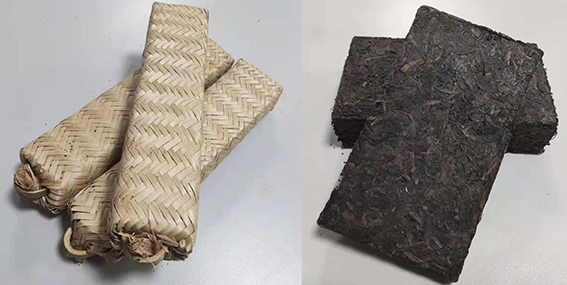

Supplement: Supplementary Figure 1 — Sichuan South-road Dark Tea. [file Image_1.TIF]

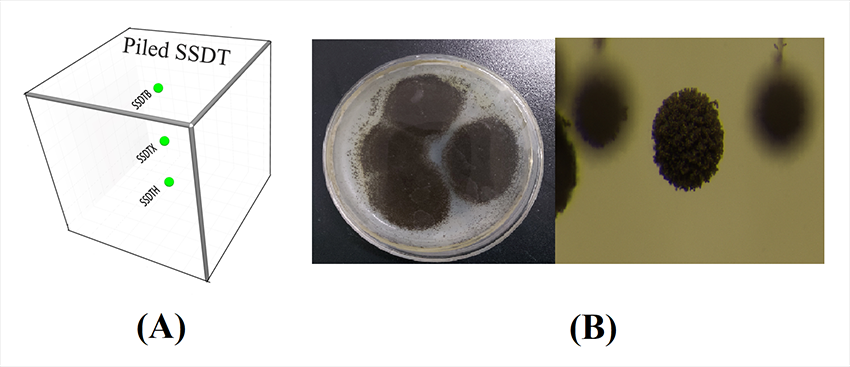

Supplement: Supplementary Figure 2 — Sampling schematic diagram and Aspergillus niger M10 strain isolated from SSDTX. (A) Sampling point. (B) Morphological characteristics of Aspergillus niger M10. [file Image_2.TIF]
